# Editorial: Contagious Risks: Perception, Behavior, and Management - Lessons From the COVID-19 Pandemic

**DOI:** 10.3389/fpsyg.2021.835088

**Published:** 2022-01-26

**Authors:** Eyal Ert, Tigran Melkonyan, Stefan T. Trautmann

**Affiliations:** ^1^Department of Environmental Economics and Management, Robert H. Smith Faculty of Agriculture Food and Environment, The Hebrew University of Jerusalem, Jerusalem, Israel; ^2^Department of Economics, Finance and Legal Studies, Culverhouse College of Business, University of Alabama, Tuscaloosa, AL, United States; ^3^Department of Economics, Heidelberg University, Heidelberg, Germany

**Keywords:** risk-taking, trust, pandemic, behavioral decision research, experiments, behavior

The spread of the COVID-19 pandemic confronted citizens and policy makers with novel unexpected risks. Many features of this global crisis have been difficult to predict with some countries being severely impacted, while others less so. Travel and trade around the globe have been severely restricted, and with the implementation of extensive shutdowns in many regions, new and substantial mental, economic, and social risks have emerged.

The pandemic spread of a novel health risk and the subsequent emerging health, economic, and social risks pose complex trade-offs and demand new decisions from policy makers, medical experts, and citizens. People's risk perception and behavior become important variables to predict the pandemic spread and the public reaction to any policy measures aimed at containing it. Trust and compliance with policies and actions (wearing masks, getting vaccinated) also play a main role.

Behavioral decision research has yielded many insights into risk perception and behavior in a plethora of contexts. An example would be the distinction between decisions that are based on described risks (an expert reporting *via* media) and experienced risk (observations in people's daily life). Ambiguity about novel and unexpected health and economic risks with diverging expert views can influence decision making. Jogging at the nearest park has turned into a social dilemma. While many of these aspects have been studied in separation, they come together in an unprecedented and dynamically evolving way during the current crisis, making it hard to predict behavior and select appropriate risk management, communication, and policy responses. For example, if people underweigh rare disasters while deciding from experience but overweigh them while deciding from description (Hertwig et al., 2004; Erev et al., 2010), then when both experienced and described information is available on a new extreme risk, would they show complacency or panic (Erev et al., 2020)? And how would people update their beliefs about an event that was previously unforeseen (Becker et al., 2020)?

This Research Topic aims to assemble research focusing on the aspects of risk perception and risky behavior, and the resulting implications for risk management and policy response, specific to the dynamic contagious nature as experienced during the COVID-19 crisis. The papers submitted included empirical studies aiming to shed light on public risk perceptions, trust (often perceived as a social risk), reactions to various aspects of the pandemic, and resulting behaviors.

As expected, several articles in this Research Topic explored the role of risk perception. Iorfa et al. propose that the perception of risk may be an important channel through which knowledge of COVID-19 can influence precautionary behavior. Attema et al. elicit people's beliefs of the pandemic health risks (e.g., their own risk to catch the disease) during the lockdown in France, and calibrate them to available evidence on risk-related sociodemographic data at the time of their elicitation. They find that people were relatively less pessimistic about their chances of catching the virus at the beginning of the lockdown, yet they became more pessimistic toward its end. Guenther et al. examined the link between risk attitude, as elicited from several experimental and self-reported risk-taking tasks, and COVID-19-related risky behaviors. While known patterns were replicated in the general tasks (e.g., less risk taking among women), no systematic association was found between the risk-taking tasks and COVID-19-related risky behaviors. These findings demonstrate the challenge of measuring individual risk attitudes for predicting risk-taking behavior (Yechiam and Ert, 2011; Noussair et al., 2014; Frey et al., 2017), and the important role of both individual traits and contextual factors in such prediction contexts (Slovic, 1995; Kocher et al., 2019).

Some authors focused on the new risks imposed by lockdowns and the interplay of various factors with these risks. Hou et al. find that COVID-19 infection risk is associated with depressive symptoms in young adults during lockdowns, and that this relationship is attenuated by perseverance and social support. Sabater-Grande et al. assess the role of expectations in daily life satisfaction during lockdowns. They find that people who predict a longer lockdown period also tend to report a higher level of daily life satisfaction.

Other authors focused on the issue of trust in healthcare and policy makers. Antinyan et al. demonstrate the importance of trust in healthcare systems in low- and middle-income countries in a national online survey in Armenia. The results show that low trust in the healthcare system might facilitate the spread of the disease and increase its likelihood to progress to a severe form, by lowering the probability of seeking treatment when people experience initial symptoms. Similarly, Bertin et al. focus on the dangers of mistrust, showing that conspiracy beliefs and mentality are associated with negative attitudes toward vaccines, lower intention to get vaccinated, and supporting chloroquine as treatment for COVID-19. In an interesting related analysis, Sanders et al. evaluate public trust and acceptability of behavioral science contributions to policy making in the COVID-19 high-risk high-stakes context, in an effort to identify factors that may foster its trust and credibility. They examine salience and sentiment toward keywords such as “psychology” in both print and social media. They find more negative sentiments toward behavioral sciences when it is mentioned in a policy context, as it is typically expressed toward the policy actors. The most prominent negativity emerges for the concept “nudge,” and for policies restricting citizens' choices. A lower level of trust is also observed when the media features conflicts between different behavioral scientists.

The articles reviewed here address various questions with different methods and approaches. They provide merely a peek into the ways accumulated knowledge and insights from behavioral decision sciences could inform novel high-risks high-stakes decision making such as COVID-19. Yet they also suggest how judgements and decisions in such a complex and unique context may enrich our understanding of the factors and processes that underlie risky choices more generally. Thus, while the current Research Topic has delivered not only answers but also many new questions, we believe that posing these questions is useful to advance our understanding of risk-taking behavior.

## Author Contributions

All authors listed have made a substantial, direct, and intellectual contribution to the work and approved it for publication.

## Conflict of Interest

The authors declare that the research was conducted in the absence of any commercial or financial relationships that could be construed as a potential conflict of interest.

## Publisher's Note

All claims expressed in this article are solely those of the authors and do not necessarily represent those of their affiliated organizations, or those of the publisher, the editors and the reviewers. Any product that may be evaluated in this article, or claim that may be made by its manufacturer, is not guaranteed or endorsed by the publisher.
